# CNP-induced cGMP signaling reduces growth cone stiffness and Ca^2+^ levels in embryonic DRG neurons

**DOI:** 10.3389/fnmol.2026.1769175

**Published:** 2026-03-24

**Authors:** Aylin Balmes, Hannes Schmidt, Stefanie Peters, Selin Kenet, Adelina Botezatu, Lai Wen, Alexandra Böttcher, Peter M. Benz, Robert Feil, Tilman E. Schäffer

**Affiliations:** 1 Institute of Applied Physics, University of Tübingen, Tübingen, Germany; 2 Interfaculty Institute of Biochemistry, University of Tübingen, Tübingen, Germany; 3 Centre for Molecular Medicine, Institute for Vascular Signalling, Goethe University, Frankfurt am Main, Germany; 4 German Centre for Cardiovascular Research (DZHK), Partner Site Rhine-Main, Frankfurt am Main, Germany

**Keywords:** actin cytoskeleton, axonal branching, Ca^2+^ signaling, cGKI, cGMP, Young’s modulus, scanning ion conductance microscopy, SICM

## Abstract

A cyclic guanosine monophosphate (cGMP) signaling pathway composed of the extracellular ligand C-type natriuretic peptide (CNP), the transmembrane natriuretic peptide receptor 2 (Npr2), and the cGMP-dependent protein kinase I (cGKI) regulates axon bifurcation of embryonic dorsal root ganglion (DRG) neurons in mice. Despite the importance of this process for the development of neuronal connectivity, the underlying mechanisms are only partially understood. Axon bifurcation requires an orchestrated rearrangement of the cytoskeleton in growth cones, the highly motile structures at axon tips. In this study, we explored the effects of cGMP signaling on growth cones in fixed and living DRG explant cultures obtained from mouse embryos. The cytoskeletal organization and stiffness of growth cones was examined by fluorescence microscopy and scanning ion conductance microscopy (SICM). Activation of cGMP signaling by CNP or the membrane-permeable cGMP analog 8-Bromo-cGMP reduced growth cone and axon shaft stiffness. Experiments with DRG neurons from Npr2 knockout (KO) mice confirmed that the anti-stiffness effect of CNP was Npr2-dependent. Pharmacological disruption of the cytoskeleton revealed that growth cone stiffness was determined by F-actin content. Activation of cGMP signaling reduced F-actin content in growth cones. Next, we studied the mechanism of cGMP-mediated cytoskeletal remodeling in growth cones. Genetic deletion of vasodilator-stimulated phosphoprotein (Vasp), a phosphorylation target of cGKI that regulates actin polymerization, did not impair cGMP-induced reduction of growth cone and axon shaft stiffness *in vitro* and axon bifurcation *in vivo*. Since growth cone dynamics is also regulated by the intracellular Ca^2+^ concentration, we performed simultaneous imaging of cGMP and Ca^2+^ in living growth cones. CNP-induced cGMP elevations suppressed ATP-induced Ca^2+^ transients in wild-type growth cones, but not in cGKI-deficient growth cones. In summary, this study indicates that the CNP-Npr2-cGMP-cGKI axis in DRG neurons controls Ca^2+^ signaling, remodeling of the actin cytoskeleton, and growth cone mechanics. Thereby, it might contribute to regulating axonal branching.

## Introduction

Axonal branching is a key process in establishing the complex patterns of neuronal connectivity that enable cognitive information processing. The axonal arborization of a neuron gives rise to several axonal projections, each targeting distinct destinations. Two principal modes of axonal branching are distinguished based on their cellular location: collateral formation from the axon shaft and ramification of the growth cone at the axon’s tip, which mainly results in a bifurcation (Gallo, 2011). Despite its importance for nervous system function, the molecular cues and mechanisms that initiate or regulate axonal branching remain only partially understood.

Studies of the stereotyped afferent patterns in embryonic dorsal root ganglion (DRG) neurons revealed a cyclic guanosine monophosphate (cGMP) signaling pathway that controls axon bifurcation at the dorsal root entry zone of the embryonic mouse spinal cord (Chédotal, 2019; Dumoulin et al., 2021; Schmidt et al., 2022). This pathway involves three key components: the ligand C-type natriuretic peptide (CNP), the transmembrane cGMP-generating guanylyl cyclase natriuretic peptide receptor 2 (Npr2; also known as guanylyl cyclase B, GC-B), and the cGMP-dependent protein kinase I (cGKI) (Dumoulin et al., 2021). The cGMP-mediated branching mechanism entails bifurcation of the axonal growth cone into two daughter axons (Dumoulin et al., 2018b), thereby increasing the complexity and integrative capacity of sensory networks. Disruption of this pathway leads to pronounced morphological and functional deficits. Loss of CNP (Schmidt et al., 2009; Zhao and Ma, 2009), Npr2 (Ter-Avetisyan et al., 2014), or cGKI (Schmidt et al., 2002; Zhao et al., 2009) prevents bifurcation of central axons of DRG neurons, causing axons to project unilaterally along the dorsolateral spinal cord, either rostrally or caudally. Functionally, Npr2 ablation in DRG neurons reduces the sensitivity to noxious thermal stimuli, underscoring the importance of axon bifurcation for effective somatosensory processing (Tröster et al., 2018).

While it is well known that CNP-bound Npr2 produces the intracellular signaling molecule cGMP, which activates cGKI, a mechanistic understanding of the axon branching process also requires identifying the downstream effectors of cGKI that coordinate axonal remodeling. Recent studies have implicated cGMP signaling in the modulation of growth cone activity in neurons. CNP application was reported to increase growth cone area and promote neurite growth in cultured DRG neurons (Dumoulin et al., 2018a). Moreover, *in vitro* studies suggest that cGMP signaling influences growth cone motility and axonal branching by modulating microtubule dynamics (Akiyama et al., 2016; Dumoulin et al., 2018a; Xia et al., 2013). Yet, the direct impact of cGMP signaling on cytoskeletal architecture and mechanical properties within growth cones remains unclear.

Growth cones are highly motile structures at axon tips that integrate extracellular cues to direct axonal pathfinding. They consist of a central domain, containing axonal microtubules that spread into the growth cone, a peripheral domain, which is fringed with filamentous actin (F-actin) forming filopodia and lamellipodia, and a transition zone between them. Growth cone navigation is mediated by tightly regulated cytoskeletal dynamics, involving coordinated remodeling of F-actin, microtubules, and intermediate filaments (Gallo and Letourneau, 2004; Geraldo and Gordon-Weeks, 2009). Cytoskeletal rearrangements influence not only the morphology and motility of growth cones, but also their mechanical properties, such as their stiffness. These properties then affect the force generation and responsiveness to environmental cues (Franze and Guck, 2010; Kerstein et al., 2015; Sánchez-Huertas and Herrera, 2021). Recent findings in platelets and vascular smooth muscle cells indicate that cGMP signaling can alter cell stiffness, as determined using atomic force microscopy (AFM) and scanning ion conductance microscopy (SICM) (Balmes et al., 2024; Budbazar et al., 2025).

SICM is a versatile tool for the study of biological samples (Hansma et al., 1989; Zhu et al., 2021). In SICM imaging, the sample is scanned with an electrolyte-filled nanopipette to which a voltage is applied and the ion current through the nanopipette is measured. Although initially developed as a method to measure the topography of nonconducting surfaces (Hansma et al., 1989), SICM has been further developed to allow the measurement of mechanical properties such as stiffness (Rheinlaender and Schäffer, 2013, 2019; Sánchez et al., 2008; Kolmogorov et al., 2021). SICM has been verified to yield cellular stiffness values comparable to those obtained using AFM, a well-established and widely used method to determine the mechanical properties of cells (Rheinlaender and Schäffer, 2013). As there is no physical contact between the nanopipette and the sample, SICM is particularly well-suited for studying fragile biological samples (Schäffer, 2013). SICM has been used to visualize nanoscale dynamic structural changes in living neurons (Takahashi et al., 2020). Furthermore, it can be used to assess changes in the stiffness of neuronal somata and dendrites in response to locally administered glutamate (Kolmogorov et al., 2023). However, the stiffness of living neuronal growth cones, which are particularly fragile structures, has not previously been investigated using SICM.

We hypothesized that CNP-Npr2-cGMP-cGKI signaling alters the mechanical properties of DRG neuron growth cones through cytoskeletal rearrangements. In this study, we investigated whether CNP-induced cGMP signaling affects growth cone stiffness and the underlying cytoskeletal organization in DRG neurons using SICM and fluorescence microscopy of living and fixed growth cones, respectively. We also explored the relationship between cGMP signals and intracellular Ca^2+^ levels by simultaneous live-cell imaging of cGMP and Ca^2+^ in growth cones.

## Materials and methods

### Mice

To measure growth cone stiffness, explant cultures were prepared from embryonic DRGs of C57BL/6, Npr2^*lacZ*/*lacZ*^ [B6.129P2-Npr2^TM1.1(*nlsLacZ*)/*Fgr*^] (Ter-Avetisyan et al., 2014) and Vasp^*KO*/*KO*^ mice (C57BL/6-Vasp^TM1*Mzim*^/Apb) (Hauser et al., 1999). Since Npr2^*lacZ*/*lacZ*^ females are infertile and many homozygous mutants do not survive to weaning, Npr2^*lacZ*/*lacZ*^ embryos were generated by crossing heterozygous Npr2^*wt*/*lacZ*^ males and females. DRGs from individual embryos were dissected separately, and genotyping was performed by PCR prior to SICM measurements. For Western blot analysis of Vasp phosphorylation, DRGs were isolated from embryonic C57BL/6, Npr2^*wt*/*cn*^ and Npr2^*cn*/*cn*^ mice (Npr2^*cn*^/J) (Tsuji and Kunieda, 2005). As with the Npr2^*lacZ*/*lacZ*^ line, Npr2^*wt*/*cn*^ and Npr2^*cn*/*cn*^ embryos were generated by crossing Npr2^*wt*/*cn*^ heterozygous mice. To analyze the role of Vasp in DRG axon bifurcation, Vasp^*wt*/*KO*^; Thy1^*YFP–H*^ and Vasp^*KO*/*KO*^; Thy1^*YFP–H*^ mice were generated by crossing Vasp KO mice with the Thy1-YFP-H reporter line [B6.Cg-Tg(Thy1-YFP)^*HJrs*/*J*^] (Feng et al., 2000). For FRET-based cGMP recordings in embryonic DRG explants, we used the R26-CAG-cGi500(L1) mouse line [Gt(ROSA)26Sor^TM1.1(*CAG–ECFP*/EYFP*)/*Feil*^], which globally expresses the cGMP biosensor cGi500 (Thunemann et al., 2013b). To assess the role of cGKI in potential cross-talk between cGMP and Ca^2+^ signaling, cGKI knockout (KO) mice (B6.129-Prkg1^TM2.1/*Naw*^) (Wegener et al., 2002) were crossed with R26-CAG-cGi500(L1) mice. Genotyping of transgenic mouse lines was performed using primers described in the corresponding original publications. All mouse lines were maintained on a C57BL/6 genetic background. Mice were housed under a 12-h light/dark cycle with *ad libitum* access to food and water. All animal procedures were approved by the relevant governmental authorities (Regierungspräsidium Tübingen, Germany, and LaGeSo Berlin, Germany).

### DRG explant cultures

DRGs were dissected together with the spinal cord from embryonic day (E)12.5 mouse embryos as described previously (Schmidt and Rathjen, 2011). The isolated spinal cords were transferred to ice-cold Hanks’ balanced salt solution (HBSS) (Thermo Fisher Scientific, 24020091), and DRGs were separated from the spinal cord using Vannas spring scissors. DRGs were trimmed into similarly sized explants using a fire-polished tungsten wire. For SICM imaging, DRGs were plated on plastic Petri dishes (Greiner bio-one, 627161); for fluorescence microscopy, they were cultured on glass-bottom Petri dishes (ibidi, 81218-800). Prior to use, dishes were coated with 50 μg/ml poly-D-lysine (Thermo Fisher Scientific, A3890401) for 3 h at 37 °C, followed by 20 μg/ml laminin (Thermo Fisher Scientific, 23017015) overnight at 37 °C. To support neurite outgrowth, explants were incubated overnight at 37 °C, 5% CO_2_, and 95% humidity in DRG growth medium consisting of Neurobasal medium (Thermo Fisher Scientific, 21103049) supplemented with 2% B-27 (Thermo Fisher Scientific, 17504044), 0.25 mM L-glutamic acid (Sigma Aldrich, G8415), 2 mM L-glutamine (Thermo Fisher Scientific, 25030081), 1% (v/v) penicillin/streptomycin (Thermo Fisher Scientific, 15140148), 50 ng/ml recombinant human NGF-β (R&D Systems, 256-GF-100), and 20 ng/ml recombinant human NT-3 (R&D Systems, 267-N3). 1 h before SICM measurements, DRG explant cultures were treated with one of the following compounds: 1 mM 8-Br-cGMP (Sigma Aldrich, B1381; 100 mM stock solution in water), 200 nM CNP (Biomol, Cay24401-500; 100 μM stock solution in water), 1 μM cytochalasin D (Sigma Aldrich, C8273; 10 mM stock solution in DMSO), or 33 μM nocodazole (Sigma Aldrich, M1404; 33 mM stock solution in DMSO). A final DMSO concentration of 0.1% (Sigma Aldrich, D8418) was used as a vehicle control where appropriate.

### SICM imaging

A custom-built SICM setup described elsewhere was used for all SICM measurements (Seifert et al., 2017). A CO_2_-laser-based puller (P-2000, Sutter Instruments) was used to fabricate borosilicate nanopipettes with a typical inner radius of 250 nm. A pressure of 10 kPa was applied to the nanopipette, leading to an indentation of the sample depending on its mechanical properties and the vertical pipette position. The ion current I was recorded versus the vertical pipette position z (I-z-curves) with a trigger setpoint of 0.98 I_0_, where I_0_ denotes the maximum ion current. The stiffness was derived from the slope of the I-z-curves between 0.99 I_0_ and 0.98 I_0_ as previously described (Rheinlaender and Schäffer, 2013).

### Data analysis and statistics of SICM imaging

SICM data were analyzed in Igor Pro (Wavemetrics). Growth cone and axon shaft regions were determined using a height threshold of 50 nm combined with manual selection. Data were tested for normality using the Shapiro–Wilk-test. Data were normally distributed except for axon shaft stiffness of wild-type (WT) neurons treated with DMSO, cytochalasin D or nocodazole, and axon shaft stiffness of Vasp KO neurons treated with 8-Br-cGMP. When all compared groups were normally distributed, they were tested using the *t*-test for comparing two groups and ANOVA and Tukey’s test for comparing more than two groups. When the data of at least one of the compared groups was not normally distributed, they were tested using the Dunn-Holland-Wolfe test. Data were considered significantly different for *p*-values < 0.05. The significance level is indicated by asterisks (*p < 0.05; **p < 0.01; ***p < 0.001; n.s. indicates no significant difference).

### Fluorescence staining

Culture medium was removed from the samples, and they were washed once with phosphate buffered saline (PBS; Sigma Aldrich, D8537). Thereafter, samples were fixed for 30 min in PBS containing 4% formaldehyde (Sigma Aldrich, F1635) and 44.4 mM D-glucose (Carl Roth, HN06.1). After washing the samples two times with PBS they were permeabilized for 10 min in PBS containing 0.1% Triton™-X-100 (Sigma Aldrich, X100). Samples were washed three times with PBS and then blocked with PBS containing 1% bovine serum albumin (BSA; Sigma Aldrich, A7906) for 10 min. Next, the samples were washed three times with PBS and stained for 30 min at room temperature using ActinGreen™ 488 ReadyProbes™ Reagent (Thermo Fisher Scientific, R37110) diluted in PBS (volume ratio 1:2000). After washing the samples three times with PBS they were stained for 1 h at room temperature in PBS containing Alexa Fluor ^®^ 594 Anti-alpha Tubulin antibody [DM1A] (abcam, ab195889) (volume ratio 200:1) and 0.1% BSA. Samples were washed three times with PBS prior to imaging with an inverted optical microscope (Nikon, Tokyo, Japan, Ti-E) using a 100×/1.45 oil objective.

### Data analysis and statistics of fluorescence staining

Fluorescence images were analyzed using Fiji (Schindelin et al., 2012). The fluorescence intensity values were normalized by growth cone area and corrected for background fluorescence intensity to obtain the mean fluorescence intensity. Data were further analyzed in Igor Pro (Wavemetrics, Portland, OR, USA). Data were tested for normality using the Shapiro–Wilk-test. Since data were typically not normally distributed, they were tested using the Dunn-Holland-Wolfe test. Data were considered significantly different for *p*-values < 0.05. The significance level is indicated by asterisks (*p < 0.05; **p < 0.01; ***p < 0.001; n.s. indicates no significant difference).

### Analysis of Vasp phosphorylation by Western blotting

The phosphorylation-dependent mobility shift of Vasp from 46 to 50 kDa was utilized as an indicator of cGKI activation. DRGs were isolated from E13.5 embryos of C57BL/6, Npr2^wt/cn^, or Npr2^cn/cn^ mice as described above for explant cultures. Thirty DRGs per sample were collected in HBSS supplemented with 100 nM each of the serine/threonine phosphatase inhibitors calyculin A (Enzo, BML-EI192) and okadaic acid (Enzo, ALX-350-003). DRGs from C57BL/6 mice were incubated for 15 min at 37 °C in HBSS, with or without 0.5 μM CNP (Merck, 05-23-0310). DRGs from Npr2^wt/cn^ and Npr2^cn/cn^ embryos were incubated either with 1 mM 8-pCPT-cGMP (Merck, C5438) for 5 min at 37 °C, or with 0.5 μM CNP for 15 min at 37 °C. Samples were then solubilized in sodium dodecyl sulfate polyacrylamide gel electrophoresis (SDS-PAGE) sample buffer and denatured at 95 °C for 5 min. Following centrifugation at 18,000 × *g* for 5 min at 4 °C, equal volumes of supernatants were separated by 10% SDS-PAGE and transferred to polyvinylidene difluoride (PVDF) membranes (Roche, 03010040001) using a blotting device (Thermo Fisher Scientific, B1000). Vasp was detected using an anti-Vasp antibody (Cell Signaling Technology, 3132) and a horseradish peroxidase (HRP)-conjugated secondary antibody followed by chemiluminescence analysis with WesternBright™ Sirius HRP Substrate (Biozym, K-12043-D20) on a ChemiDoc MP Imaging System (Bio-Rad, 12003154). Relative pixel intensities of the 50 kDa (phosphorylated at Ser 153 of murine Vasp) and 46 kDa (unphosphorylated at Ser 153 of murine Vasp) Vasp bands were quantified using Fiji (Schindelin et al., 2012).

### Analysis of axon bifurcation

To assess the effect of Vasp deletion, the morphology of DRG neurons was examined in Vasp^wt/KO^; Thy1^YFP–H^ and Vasp^KO/KO^; Thy1^YFP–H^ mice, which express yellow fluorescent protein (YFP) under control of the Thy1 promoter in a subset of DRG neurons. Spinal cords were dissected from postnatal day (P)15 mice and fixed in 4% paraformaldehyde in PBS for 4 h at room temperature. Samples were then washed three times in PBS. After mounting, the branching patterns of central axons of DRG neurons at the dorsal root entry zone were analyzed using a confocal laser scanning microscope (Zeiss LSM 710). To visualize individual axonal trajectories, Z-stacks were acquired at 1 μm intervals using 514 nm laser excitation and detection of fluorescence emission in the 520–550 nm range. Z-stacks were subsequently processed into maximum intensity projections using Fiji (Schindelin et al., 2012). Only labeled axons that could be clearly identified as individual axons were included in the analysis. All analyses were performed blinded to genotype.

### Ca^2+^/cGMP imaging

Ca^2+^ and FRET/cGMP imaging were performed on DRG explant cultures prepared from E12.5 R26-CAG-cGi500(L1) mice expressing the genetically encoded cGMP sensor cGi500 (Thunemann et al., 2013b). Imaging was conducted 24 h after plating using an epifluorescence microscopy setup as previously described (Paolillo et al., 2018; Schmidt et al., 2016, 2018). The system included an inverted Axiovert 200 microscope (Zeiss) equipped with a NeoFluar 40×/1.30 oil objective, a light source with excitation filter switching device (Oligochrome; TILL Photonics GmbH), a Dual-View beam splitter (Photometrics) with a 516-nm dichroic mirror and emission filters for cyan fluorescent protein (CFP) (480/30 nm) and YFP (535/40 nm), and a charge-coupled device camera (Retiga 2000R, QImaging).

For simultaneous Ca^2+^ and cGMP imaging, DRG explants were incubated with 2.5 μM Fura-2/AM (Calbiochem; 1 mM stock solution in DMSO) diluted in Tyrode buffer (140 mM NaCl, 5 mM KCl, 1.2 mM MgSO_4_, 2.5 mM CaCl_2_, 5 mM D-glucose, 5 mM HEPES, pH 7.4) for 35 min at 37 °C in the dark prior to imaging. Coverslips were mounted in a superfusion chamber (Warner Instrument SA-20LZ, Harvard Bioscience) and continuously superfused at 21 °C with Tyrode buffer ± test compounds at a flow rate of 1 ml/min. Test compounds were applied via Pharmacia IV-7 injection valves (GE Healthcare). For Fura-2-based Ca^2+^-imaging, excitation was alternated between 340/26 nm and 387/11 nm using bandpass filters, and emission was detected at 535/40 nm. For cGMP imaging using cGi500, excitation was provided at 445/20 nm and emission was simultaneously recorded at 480/30 nm (CFP) and 535/40 nm (YFP).

### Data processing and statistical analysis of Ca^2+^/cGMP imaging

Imaging data were analyzed as previously described (Thunemann et al., 2013a,b), including procedures for signal correction and ratio calculation. VisiView software (Visitron) was used for image acquisition and online analysis, while offline analysis was performed using Fiji software (Schindelin et al., 2012). Subsequent data processing and statistical evaluation were carried out using Microsoft Excel (Microsoft Corp.) and Origin (OriginLab Corp.). For Ca^2+^ imaging, emission signals at 535 nm following excitation at 340 nm (F_340_) and 387 nm (F_387_) were used to compute the F_340_/F_387_ ratio (*R_*Ca2*+_*, black traces). Fluorescence signals at 480 nm (F_480_; CFP emission) and 535 nm (F_535_; YFP emission) were background-corrected and used to calculate the F_480_/F_535_ ratio (*R*_*cGMP*_, shown as brown traces). Changes in fluorescence (ΔF/F) and corresponding ΔR/R values were normalized to baseline levels recorded over the first 3 min of each experiment. Peak area and peak height values of ΔR/R responses were quantified using the Peak Analyzer module in Origin, with peak boundaries defined manually based on visual inspection.

Statistical analysis was performed using Origin software. Comparison between more than two groups were performed by one-way ANOVA followed by Bonferroni’s *post-hoc* test. Results were considered statistically significant at *p* < 0.05.

#### Quantification of growth cone filopodia

To assess the effects of CNP and ATP on growth cone dynamics, filopodia per growth cone were quantified in DRG explant cultures derived from E12.5 C57BL/6 mouse embryos. Three independent experiments were performed with 3–4 explants per condition. Explants were treated with (1) Neurobasal medium (Thermo Fisher Scientific, 21103049) as control, (2) 200 nM CNP (Biomol, Cay24401-500), (3) 100 μM ATP (AppliChem, A1348), or (4) 200 nM CNP plus 100 μM ATP.

Silicone grids from Permanox chambers (Thermo Fisher Scientific, 177402) were mounted onto Lumox dishes (Sarstedt, 94.6077.410) before coating with poly-D-lysine and laminin (see section “DRG explant cultures”), generating six wells per dish. One DRG explant was plated per well in 125 μl DRG growth medium. After 20 h, 10 μl of Neurobasal medium (conditions 1 and 3) or 10 μl of 2.7 μM CNP diluted in Neurobasal medium (final concentration of 200 nM CNP; conditions 2 and 4) was added. After 15 min pre-incubation, 10 μl of Neurobasal medium (conditions 1 and 2) or 10 μl of 1.45 mM ATP (final concentration of 100 μM ATP; conditions 3 and 4) was applied, followed by 60 min incubation at 37 °C and 6% CO_2_. For fixation, 70 μl of culture medium was gently removed from each well and replaced with 75 μl of 4% formaldehyde (Sigma-Aldrich, F1635) supplemented with 44.4 mM D-glucose (Carl Roth, HN06.1). Cultures were fixed for 15 min at room temperature and washed three times with cold PBS. Blocking was performed for 30 min in PBS containing 1% bovine serum albumin (BSA; Carl Roth, 8076.4). Delicate filopodial structures were visualized by immunofluorescence using a rabbit anti-L1CAM (0.5 μg/ml in blocking solution; gift from Fritz G. Rathjen, MDC Berlin) followed by Cy3-conjugated goat anti-rabbit IgG (Jackson ImmunoResearch, 111-165-144; 1:1000). Cultures were stored in 50% glycerol in PBS at 4 °C until imaging.

Growth cones were imaged using a confocal laser scanning microscope (LSM 710 or LSM 980, Zeiss) with a 20×/0.8 objective. Filopodia were defined as thin growth cone protrusions measuring 1–12 μm in length and exhibiting no more than three branches. Structures meeting these criteria were semi-automatically traced using the Skeletonize function of the Simple Neurite Tracer plugin (Longair et al., 2011) in Fiji (Schindelin et al., 2012). For each independent experiment, filopodia counts per growth cone were normalized to the mean control value. Statistical analyses were performed using GraphPad Prism version 9.0.0 (GraphPad Software). Groups were compared using the Kruskal–Wallis test with Dunn’s *post-hoc* test.

## Results

### CNP reduces DRG growth cone stiffness via Npr2

**FIGURE 1:**
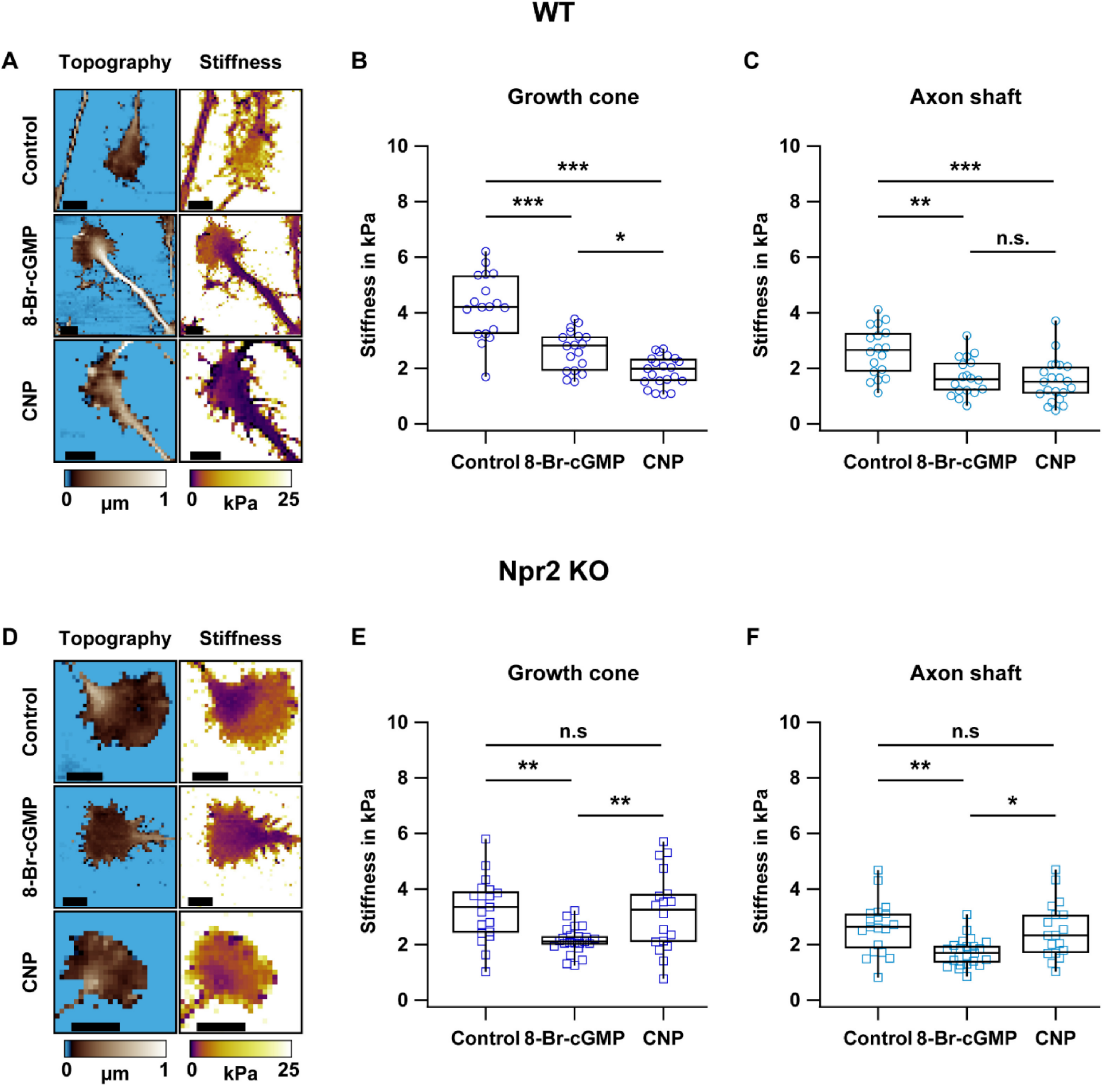
SICM measurements reveal that activation of the CNP-Npr2-cGMP axis reduces the stiffness of DRG growth cones. **(A,D)** Representative SICM-derived topography and stiffness images of living DRG growth cones from WT **(A)** and Npr2 KO **(D)** embryos under control conditions or after treatment with 1 mM 8-Br-cGMP or 200 nM CNP. Treatments were applied 1 h prior to SICM imaging. Scale bars, 10 μm. **(B,C,E,F)** Quantification of stiffness in WT **(B)** and Npr2 KO **(E)** growth cones as well as WT **(C)** and Npr2 KO **(F)** axon shafts. Each data point represents the median stiffness of a single growth cone or axon shaft. For each condition, 18–23 growth cones and axon shafts were analyzed from 3 to 4 independent DRG explants. The significance level is indicated by asterisks (*p < 0.05; **p < 0.01; ***p < 0.001; n.s. indicates no significant difference).

To assess whether CNP-induced cGMP signaling affects the mechanical properties of DRG growth cones, DRG explant cultures from E12.5 WT embryos were treated with either 1 mM 8-Br-cGMP, a membrane-permeable cGMP analog that activates cGKI, or 200 nM CNP, the ligand for the cGMP-producing receptor guanylyl cyclase Npr2. After 1 h of treatment, growth cones were analyzed using SICM. This technique provided high-resolution images of both surface topography and mechanical stiffness of individual growth cones (Figure 1A). SICM imaging revealed that the central domain of DRG neuronal growth cones was typically thicker (indicated by brighter colors in topography images) compared to the peripheral domain. Treatment with 8-Br-cGMP or CNP significantly reduced the median stiffness of growth cones from ≈4.2 kPa under control conditions to ≈2.8 kPa or ≈2.0 kPa, respectively (Figure 1B). Similarly, treatment with 8-Br-cGMP or CNP also led to a significant reduction in axon shaft stiffness from ≈2.7 kPa under control conditions to ≈1.6 kPa or ≈1.5 kPa, respectively (Figure 1C). These findings indicated that CNP-induced cGMP signaling decreases the mechanical stiffness of DRG growth cones and axon shafts, presumably via remodeling of the growth cone cytoskeleton.

To test whether the stiffness-reducing effect of CNP was mediated by the cGMP-producing CNP receptor Npr2, similar experiments were conducted with DRG explant cultures from E12.5 Npr2 KO embryos (Figure 1D). Notably, compared to untreated controls, CNP treatment did not reduce the stiffness of Npr2-deficient growth cones and axon shafts, whereas 8-Br-cGMP, which acts downstream of Npr2, still effectively decreased stiffness (Figures 1E, F). These findings confirmed that the CNP-induced reduction in stiffness requires functional Npr2 signaling and an increase of cGMP.

### F-actin content determines growth cone stiffness and is reduced by cGMP signaling

**FIGURE 2:**
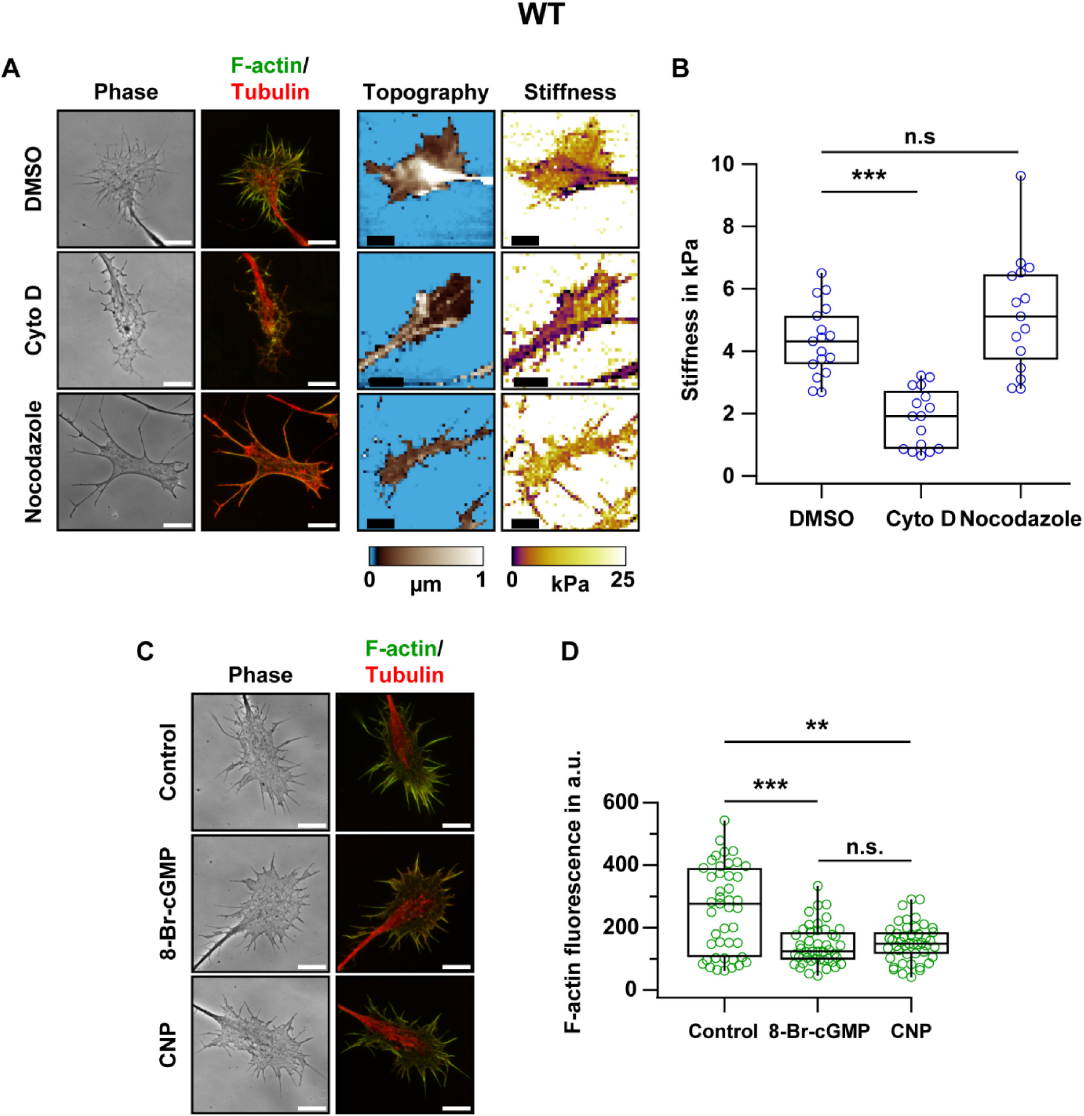
Disruption of the actin cytoskeleton, but not microtubule depolymerization, reduces growth cone stiffness, and cGMP reduces F-actin content in growth cones. **(A)** Representative images of phase contrast microscopy, fluorescence staining of F-actin (green) and tubulin (red), topography, and stiffness of WT DRG growth cones treated with 0.1% DMSO, 1 μM cytochalasin D (Cyto D), or 33 μM nocodazole. Treatments were applied for 1 h and then cells were fixed for fluorescence staining, or SICM measurements were performed to determine the stiffness of living growth cones. **(B)** SICM-based quantification of median stiffness of individual WT growth cones. Each data point represents one growth cone; 13–17 growth cones from 2 to 3 independent DRG explant cultures were analyzed per condition. **(C)** Representative phase contrast and fluorescence images of WT growth cones that were either untreated (control) or treated with 1 mM 8-Br-cGMP or 200 nM CNP for 1 h prior to fixation. F-actin and tubulin were stained in green and red, respectively. **(D)** Quantification of background-corrected F-actin fluorescence intensity in individual growth cones normalized by growth cone area. Each data point represents one growth cone; 30 growth cones from 3 independent DRG explant cultures were analyzed per condition. Scale bars, 10 μm. The significance level is indicated by asterisks (**p < 0.01; ***p < 0.001; n.s. indicates no significant difference).

To investigate the role of cytoskeletal components in growth cone stiffness, we selectively disrupted the F-actin or microtubule cytoskeleton in cultured DRG neurons. Cytochalasin D, an inhibitor of actin polymerization, was used to target the actin cytoskeleton, whereas nocodazole, which disrupts microtubule polymerization, was applied to interfere with microtubules. Both treatments altered growth cone morphology as revealed by changes in shape and structural organization (Figure 2A). Interestingly, compared to DMSO-treated controls (median stiffness ≈4.3 kPa), only cytochalasin D significantly reduced growth cone stiffness (median stiffness ≈1.9 kPa), while nocodazole had no significant effect (median stiffness ≈5.1 kPa) (Figure 2B).

Fluorescence staining revealed a marked reduction in F-actin content in WT growth cones following treatment with 8-Br-cGMP or CNP, with median fluorescence intensities of ≈120 arbitrary units (a.u.) and 150 a.u., respectively, compared to ≈280 a.u. in untreated controls (Figures 2C, D). These findings suggested that CNP-induced cGMP signaling modulates the organization of the actin cytoskeleton in DRG growth cones, thereby contributing to the observed changes in growth cone stiffness.

### Loss of Vasp does not impair axon bifurcation and cGMP effects on growth cone stiffness

**FIGURE 3:**
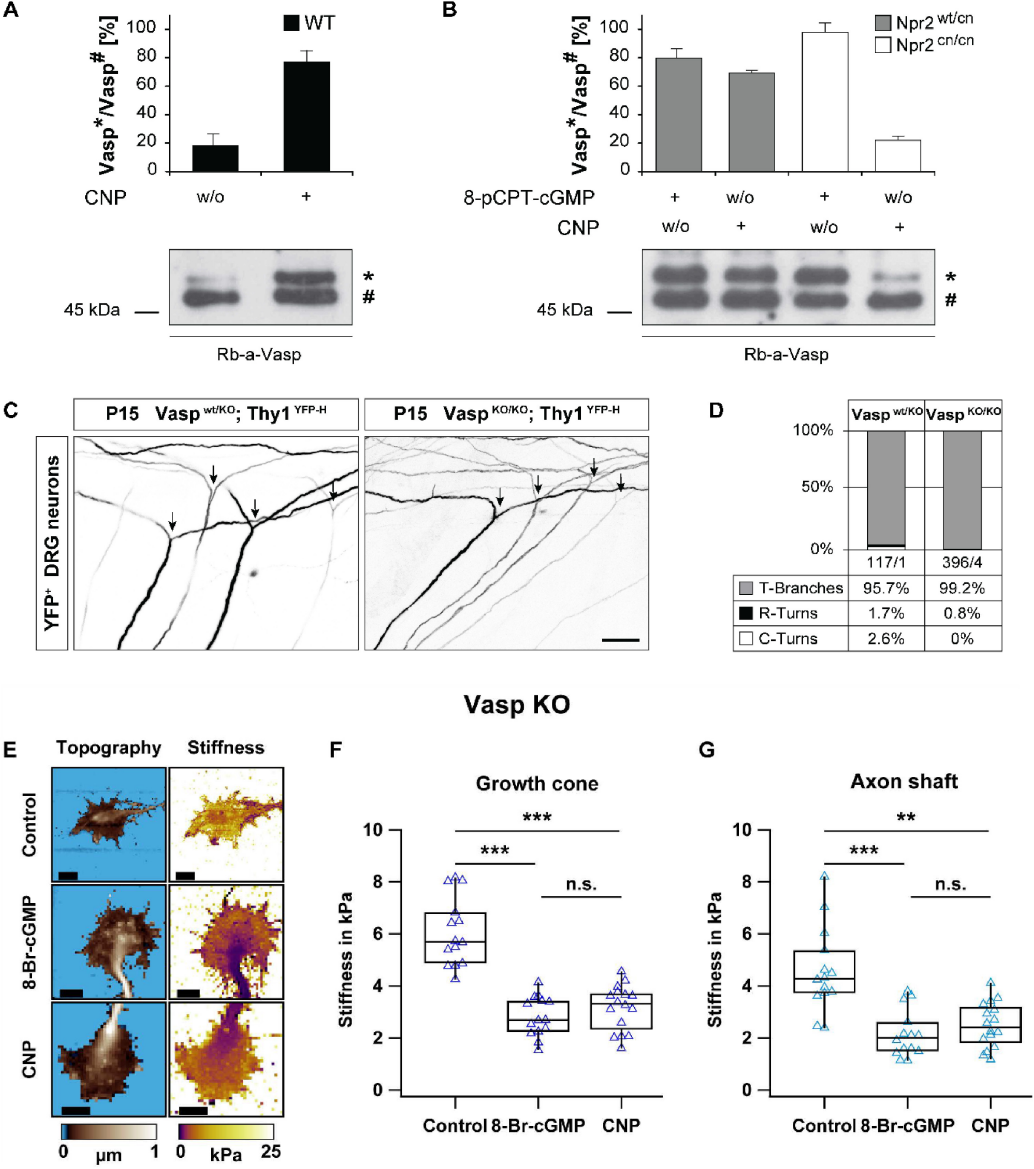
Loss of Vasp does not impair axon bifurcation and cGMP-induced reduction in growth cone stiffness in embryonic DRG neurons. **(A)** WT DRGs were stimulated with 0.5 μM CNP for 15 min. **(B)** DRGs from Npr2^wt/cn^ and Npr2^cn/cn^ mice were stimulated with CNP (0.5 μM, 15 min) or 8-pCPT-cGMP (1 mM, 5 min). A phosphorylation-dependent mobility shift of Vasp from 46 to 50 kDa, detected by Western blot analysis, was used as a readout for cGKI activation in DRGs isolated from E13.5 mouse embryos. Lower panels show representative Western blot images, and upper panels the quantification of the 50 kDa (*) and 46 kDa (#) Vasp bands, which represent Vasp phosphorylated and not phosphorylated at S153, respectively. Data represent mean ± SD [*n* = 3 for panel **(A)**, *n* = 2 for panel **(B)**]. **(C)** Analysis of axon bifurcation in DRG neurons from Vasp^wt/KO^ and Vasp^KO/KO^ mice expressing the Thy1-YFP-H reporter. Scale bar, 50 μm. **(D)** Quantification of central axon trajectories showing bifurcations (T-Branches), rostral turns (R-Turns), and caudal turns (C-turns) in DRG neurons from Vasp^wt/KO^ and Vasp^KO/KO^ mice. The number of axons/embryos analyzed is also indicated in the figure. **(E)** Representative SICM topography and stiffness images of growth cones from Vasp KO DRG explant cultures under control conditions or following treatment with 1 mM 8-Br-cGMP or 200 nM CNP for 1 h. Scale bars, 10 μm. **(F,G)** Quantification of the stiffness of Vasp KO growth cones **(F)** and axon shafts **(G)**. Each data point represents the median stiffness of a single growth cone or axon shaft. For each condition, 14–16 growth cones and axon shafts from 3 to 4 independent explant cultures were analyzed. The significance level is indicated by asterisks (**p < 0.01; ***p < 0.001; n.s. indicates no significant difference).

Vasp, a well-known substrate of cGKI, might link cGMP signaling to remodeling of the actin cytoskeleton. It is a member of the conserved enabled (Ena)/Vasp family of actin-regulatory proteins that localize to the leading edge of lamellipodia and to the tips of filopodia in neuronal growth cones (Dent et al., 2011; Lanier et al., 1999). To investigate the role of Vasp as a downstream mediator of the CNP-Npr2-cGMP-cGKI pathway in growth cones, we examined Vasp phosphorylation in embryonic mouse DRGs and its effects on axon branching and growth cone stiffness (Figure 3).

Phosphorylation of Vasp by cGKI at S153, S235, and T274 in mice (S157, S239, and T278 in humans) has been linked to the regulation of actin filament dynamics and cytoskeletal remodeling (Benz et al., 2009; Döppler and Storz, 2013; Zhuang et al., 2004). We utilized the phosphorylation-induced mobility shift of Vasp from 46 to 50 kDa, when it is phosphorylated at S153, to monitor cGKI activation. Vasp was detected in lysates of E13.5 DRGs, a developmental stage when bifurcation of the central axons of DRG neurons occurs. Treatment of WT DRGs with CNP significantly increased the 50/46 kDa Vasp ratio, indicating enhanced phosphorylation (Figure 3A). In contrast, this effect was absent in Npr2^cn/cn^ DRGs that lack functional Npr2 receptors for CNP, demonstrating that CNP-induced Vasp phosphorylation depends on Npr2. As expected, direct activation of cGKI, which acts downstream of Npr2, with the cGMP analog 8-pCPT-cGMP induced Vasp phosphorylation in both Npr2^wt/cn^ and Npr2^cn/cn^ DRGs (Figure 3B). These results showed that activation of the CNP-Npr2-cGMP-cGKI axis leads to increased Vasp phosphorylation in murine DRG neurons.

To assess the functional role of Vasp in axon bifurcation, we crossed Vasp KO mice with the Thy1-YFP-H reporter line (Feng et al., 2000) to visualize axon trajectories of individual DRG neurons. Central axon bifurcation appeared normal in both Vasp^wt/KO^ and Vasp^KO/KO^ mice (Figures 3C, D). Treatment with 1 mM 8-Br-cGMP or 200 nM CNP significantly reduced stiffness in Vasp KO growth cones (≈2.7 kPa and ≈3.3 kPa, respectively) as compared to vehicle-treated growth cones (≈5.7 kPa) (Figures 3E, F), mirroring the effects observed in WT neurons. Similarly, treatment of Vasp KO growth cones with 8-Br-cGMP or CNP also led to a significant reduction in axon shaft stiffness from ≈2.6 kPa under control conditions to ≈1.7 kPa or ≈2.3 kPa, respectively. These findings showed that although Vasp is phosphorylated in response to cGMP signaling in DRG neurons, it is apparently not required for cGMP-mediated axon bifurcation and reduction in growth cone and axon shaft stiffness. Given the potential redundancy among Ena/Vasp family members (Menzies et al., 2004), combinatorial knockout studies may be necessary to fully elucidate the collective role of these proteins in regulating axon branching and cytoskeletal mechanics.

### Ca^2+^/cGMP imaging reveals that CNP-induced cGMP signaling via cGKI suppresses ATP-evoked Ca^2+^ transients in growth cones

**FIGURE 4:**
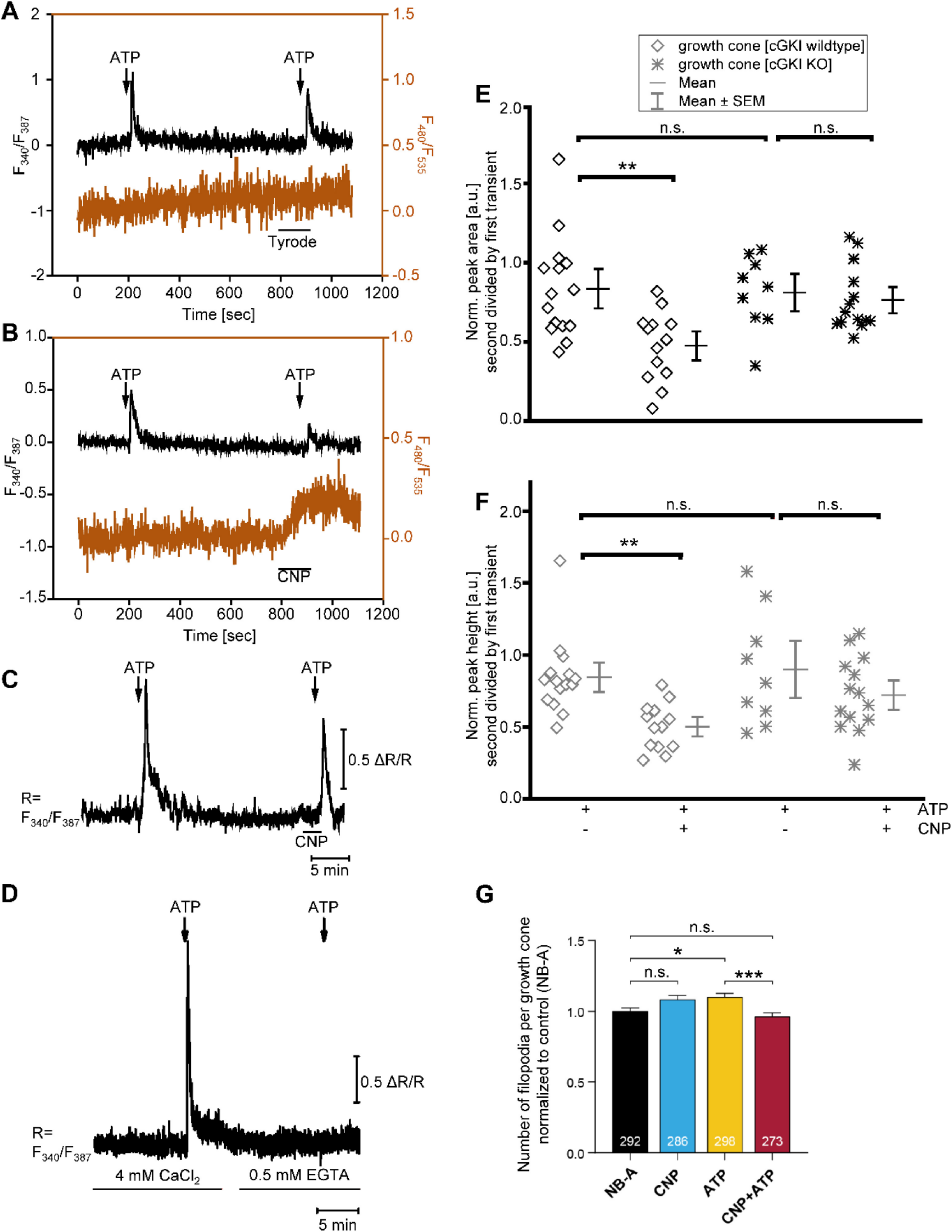
CNP-induced cGMP signaling via cGKI suppresses ATP-evoked Ca^2+^ transients and filopodia number in DRG growth cones. Simultaneous Ca^2+^ and cGMP imaging was performed in growth cones of embryonic DRG explant cultures from transgenic mouse embryos expressing the FRET-based cGMP sensor cGi500. **(A,B)** Growth cones were analyzed using Fura-2-based Ca^2+^ imaging (F_340_/F_387_ ratio, black traces) and FRET-based cGMP imaging (F_480_/F_535_ ratio, brown traces). DRG explants from WT (cGKI-expressing) embryos were subjected to two consecutive applications of ATP (100 μM). Prior to the second application, either Tyrode buffer **(A)** or CNP (100 nM) **(B)** was applied. **(C)** Ca^2+^ imaging was conducted in growth cones from cGKI KO embryos in the absence and presence of CNP. **(D)** ATP-induced Ca^2+^ transients in WT growth cones in the presence (CaCl_2_) and absence (EGTA) of extracellular Ca^2+^. For these measurements, Tyrode buffer was prepared without Ca^2+^ and then supplemented with 4 mM CaCl_2_ or 0.5 mM EGTA as indicated. Data are representative for *n* = 9 growth cones from 2 embryos. **(E,F)** Quantification of ATP-induced Ca^2+^ transients (Fura-2 ratio) was performed by measuring peak area **(E)** and peak height **(F)**, comparing responses with and without CNP treatment in growth cones from WT and cGKI KO embryos. Responses to the second ATP application were normalized to the first peak value for each individual growth cone. Each data point represents a single growth cone; *n* = 9–15 growth cones from 5 to 7 embryos were analyzed per condition. **p < 0.01; n.s., not significant. **(G)** Filopodia number per growth cone. DRG explants were grown for 20 h and then treated either with Neurobasal medium (NB-A) as a control, 200 nM CNP, 100 μM ATP, or 200 nM CNP and 100 μM ATP. Then, DRG neurons were fixed, stained, and the number of filopodia per growth cone was determined. Data was normalized to the NB-A control. Numbers within bars represent number of analyzed growth cones. Data were pooled from three independent experiments. *p ≤ 0.05; ***p ≤ 0.001; n.s., not significant.

To investigate a potential cross-talk between cGMP and Ca^2+^ signaling, we analyzed growth cones of DRG explant cultures isolated from E12.5 R26-CAG-cGi500(L1) mice expressing the cGMP biosensor cGi500 (Thunemann et al., 2013b). Explants from cGMP sensor mice were loaded with the Ca^2+^-sensitive dye Fura-2/AM, enabling simultaneous imaging of both cGMP and Ca^2+^ in individual living growth cones (Figures 4A, B; brown and black traces, respectively). Ca^2+^ transients were elicited by two sequential applications of 100 μM ATP. ATP did not change the cGMP concentration in growth cones. To elevate intracellular cGMP levels, explants were superfused with 100 nM CNP for 1.5 min before the second ATP stimulus was applied. Explants treated with Tyrode buffer instead of CNP served as controls, and as expected, Tyrode buffer did not alter Ca^2+^ or cGMP levels (Figure 4A). In contrast, application of CNP elevated cGMP in growth cones (Figure 4B). Using a micropipette for drug delivery, we observed that after local application of CNP at the axon tip, the cGMP concentration initially increased in the growth cone and then spread along the axon to the soma of the neuron (Supplementary Video 1). CNP suppressed ATP-induced Ca^2+^ transients in WT growth cones (Figure 4B) but not in growth cones lacking cGKI, the cGMP effector in DRG neurons (Figure 4C). Experiments in the presence and absence of extracellular Ca^2+^ revealed that the ATP-induced Ca^2+^ transients in growth cones required the presence of extracellular Ca^2+^ (Figure 4D). Quantification of the Ca^2+^ transient peak areas and heights (Figures 4E, F) confirmed a significant reduction of ATP-evoked Ca^2+^ transients by CNP-induced cGMP in WT growth cones but not cGKI KO growth cones. These findings identified an interaction of cGMP and Ca^2+^ signaling in DRG growth cones, whereby activation of the CNP-Npr2-cGMP-cGKI axis lowers the ATP-induced Ca^2+^ elevation. To evaluate the possibility that modulation of Ca^2+^ signaling might provide a mechanistic link to cGMP-mediated actin cytoskeleton remodeling and reduction of growth cone stiffness, we investigated whether ATP and CNP had an influence on growth cone morphology. Interestingly, treatment with ATP significantly increased the number of filopodia per growth cone, and this effect was blunted by co-application of CNP (Figure 4G).

## Discussion

In this study, we used SICM to quantify the stiffness of living neuronal growth cones. For untreated WT DRG neurons, we found a growth cone stiffness of around 4 kPa. This is in good agreement with a stiffness of 4.1±2.4 kPa, which was reported for neuronal growth cones measured using AFM (Sajid, 2023). Qualitatively, our SICM images show a higher stiffness in the peripheral domain compared to the central domain which is also in accordance with a study that used AFM stiffness mapping to investigate neuronal growth cones (Xiong et al., 2009). Overall, these data show that SICM is a suitable method to investigate growth cone stiffness.

Our SICM measurements demonstrated that CNP-induced cGMP signaling reduces growth cone stiffness in DRG neurons. This cGMP-mediated mechanical remodeling of the growth cone might contribute to axonal bifurcation of DRG neurons. For example, cell stiffness is correlated with traction forces (Rheinlaender et al., 2021) and traction forces are driving growth cone motility (Franze, 2020). Changes in local traction forces can lead to a directional change in microtubule polymerization and thereby axon growth (Franze, 2020). However, to assess how reduced growth cone stiffness might affect axonal bifurcation, further research will be necessary. Mechanistically, as shown in the present study, the reduction in stiffness may result from an interaction between cGMP-cGKI and Ca^2+^ signaling that leads to a restructuring of the actin cytoskeleton in the growth cone. An increase of cGMP resulted in reduced growth cone stiffness and F-actin content. Disruption of the actin cytoskeleton with cytochalasin D also reduced growth cone stiffness consistent with AFM measurements reported by van Tartwijk et al. (2024). Given that actin remodeling is a key regulator of growth cone motility and axon guidance (Gallo and Letourneau, 2004; Omotade et al., 2017), it is also likely involved in cGMP-dependent axon bifurcation. Application of nocodazole, which disrupts microtubules, did not affect growth cone stiffness in our experiments. This finding is consistent with a previous study reporting that nocodazole did not affect elasticity maps of living neurons measured by AFM (Spedden et al., 2012). Together, these results indicate that the anti-stiffness effect of cGMP is mainly related to an effect on the actin cytoskeleton rather than on microtubules. However, our data do not rule out the possibility that cGMP signaling in DRG neurons can also modulate microtubule dynamics (Akiyama et al., 2016; Dumoulin et al., 2018a; Xia et al., 2013).

The phosphorylation targets of cGKI that mediate alterations of the actin cytoskeleton, reduction of growth cone stiffness, and axon bifurcation of DRG neurons remain unidentified. In an attempt to explore this, we examined Vasp, a well-known cGKI substrate and regulator of the actin cytoskeleton (Dent et al., 2011). DRG neurons from Vasp KO mice exhibited normal axon bifurcation and stiffness reduction in response to 8-Br-cGMP or CNP, showing that phosphorylation of Vasp by cGKI is not required for cGMP-mediated axon bifurcation and remodeling of growth cone mechanics. However, compensation of Vasp functions in Vasp KO mice by other members of the Ena/Vasp family, such as mammalian enabled (Mena) or Ena-Vasp-like (EVL), cannot be ruled out.

The modulation of the actin cytoskeleton in growth cones involves a complex interplay of signaling pathways and second messengers including cyclic nucleotides and Ca^2+^ (Tojima et al., 2011). A potential mechanism underlying changes in stiffness observed after 8-Br-cGMP or CNP treatment may involve alterations in Ca^2+^ signaling. To test this, we performed simultaneous Ca^2+^ and cGMP imaging in living DRG growth cones and found that CNP-induced cGMP suppressed ATP-evoked Ca^2+^ transients via activation of cGKI. Ca^2+^ signaling is a well-known regulator of growth cone morphology and motility during axonal growth and guidance (Gasperini et al., 2017; Gomez and Zheng, 2006). Ca^2+^ regulation of actin dynamics promotes filopodia formation, extension, and stabilization. Indeed, our results support a link between Ca^2+^ regulation via the CNP-Npr2-cGMP-cGKI axis and remodeling of the growth cone cytoskeleton that translates into altered growth cone behavior as reflected by the number of filopodia. Future studies are required to prove that this signaling mechanism is also relevant for triggering axon bifurcation *in vivo*.

Given that cellular stiffness is strongly influenced by actin organization, cGMP-induced suppression of Ca^2+^ signaling and subsequent alterations of the actin cytoskeleton could contribute to the observed changes in growth cone stiffness. A similar mechanism may also be involved in the inhibition of semaphorin 3A-induced growth cone collapse by cGMP-cGKI signaling (Schmidt et al., 2002). This is all the more plausible given that activation of the cGMP-cGKI axis is known to lower the Ca^2+^ concentration in vascular smooth muscle cells (Feil et al., 2002) and platelets (Wen et al., 2018). There is an extensive literature describing how cGMP-cGKI signaling can affect Ca^2+^ signaling by the regulation of Ca^2+^ release from intracellular stores and/or Ca^2+^ influx via ion channels in the plasma membrane (Hofmann et al., 2006; Lincoln et al., 1994). Our findings suggest that the CNP-Npr2-cGMP-cGKI axis suppresses ATP-induced Ca^2+^ increases in neuronal growth cones mainly via inhibiting Ca^2+^ influx from the extracellular space. Interestingly, gain of function variants of cGKI in humans have been linked to aortic aneurysm and dissection, possibly because the vascular smooth muscle cells of these patients are more deformable and mechanically compromised due to an impaired actin cytoskeleton (Guo et al., 2013; Jost et al., 2026). It would be interesting to know if these patients expressing cGKI variants with increased kinase activity do also show alterations of axon bifurcation in the spinal cord.

**FIGURE 5:**
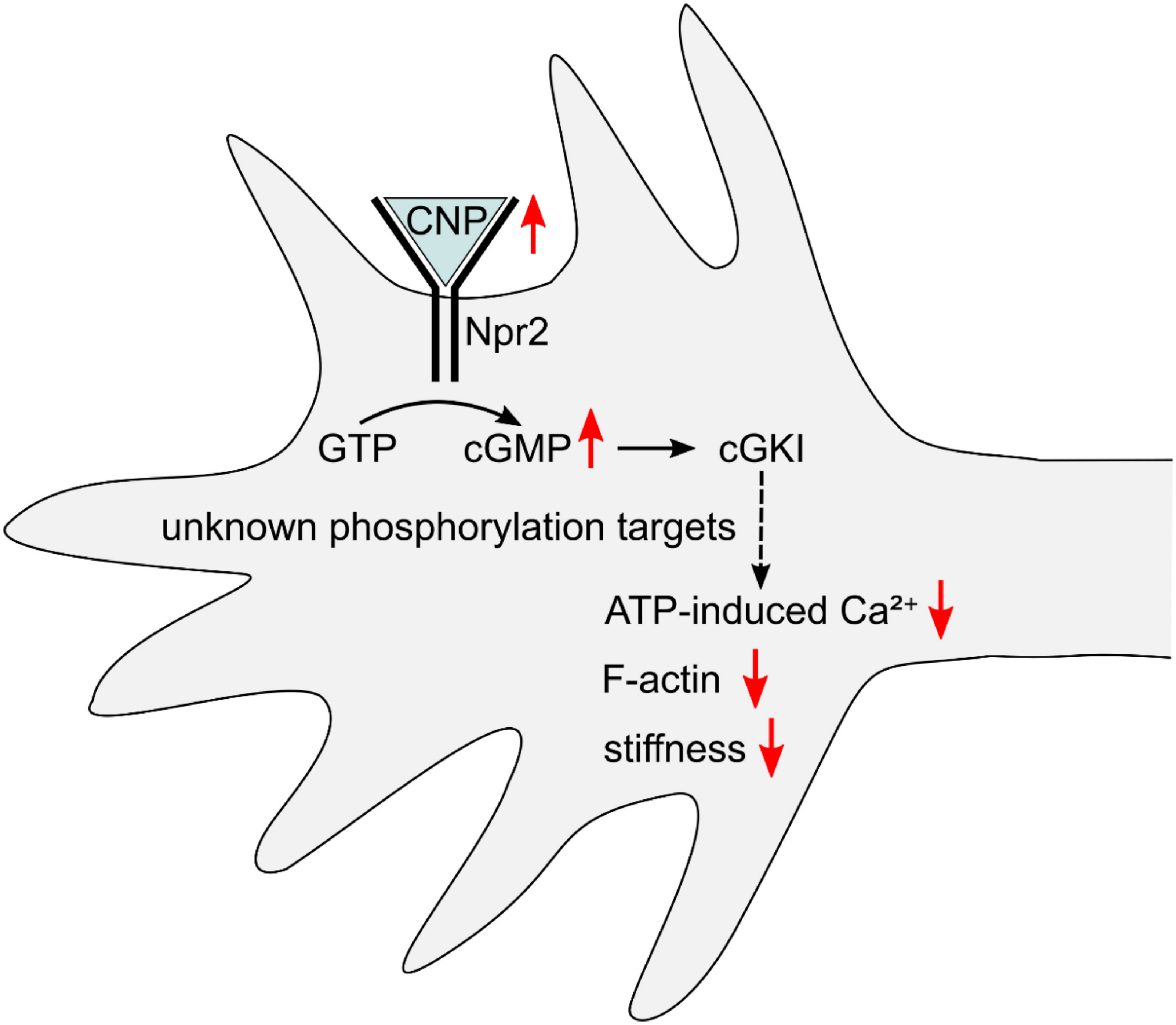
Schematic overview of the effect of CNP-Npr2-cGMP-cGKI signaling on growth cone Ca^2+^, F-actin, and stiffness of embryonic DRG neurons. For details, see main text.

Based on the previous and present data, we propose the following model for cGMP-triggered axon bifurcation (Figure 5): CNP activates Npr2, which leads to an increase of cGMP in the growth cone and activation of cGKI. The protein kinase phosphorylates one or more as-yet-unknown substrate proteins, resulting in reduced Ca^2+^ signaling, F-actin content, and stiffness, and ultimately in bifurcation of the growth cone. In the future, it will be important to identify the relevant cGKI targets that contribute to remodeling of the growth cone actin cytoskeleton. Moreover, it will be of great interest to investigate whether the cGMP/Ca^2+^/actin cross-talk identified in DRG neurons also occurs in other cell types whose stiffness is reduced by an increase of cGMP, such as platelets and vascular smooth muscle cells (Balmes et al., 2024; Budbazar et al., 2025). We anticipate that refinement of cGMP imaging methods (Feil et al., 2022; Russwurm and Koesling, 2018) will be very helpful in further improving our understanding of cGMP signaling in the regulation of growth cone stiffness. For example, by combining membrane-bound and cytosolic cGMP biosensors (Wolters et al., 2025), the role of local membrane-associated versus global cytosolic cGMP pools could be investigated. Moreover, combining optical and mechanical imaging in living cells could make it possible to directly “watch” the dynamic interplay between cGMP, Ca^2+^, the actin cytoskeleton, and growth cone mechanics in real time.

## Data Availability

The raw data supporting the conclusions of this article will be made available by the authors, without undue reservation.

## Supplementary material

Supplementary Video 1 Local stimulation of cGMP signals in DRG growth cones. CNP was applied via a glass pipette positioned at the axon tip of a DRG neuron expressing the cGMP biosensor cGi500. 1 μM CNP was ejected at 20 hPa for 30 s by the pipette. A lighter color indicates a higher cGMP concentration. Scale bar, 20 μm. Time: h:m:s.
